# Endometrial stromal sarcoma: a population-based analysis

**DOI:** 10.1038/sj.bjc.6604527

**Published:** 2008-09-23

**Authors:** J K Chan, N M Kawar, J Y Shin, K Osann, L-m Chen, C B Powell, D S Kapp

**Affiliations:** 1 Division of Gynecologic Oncology, Department of Obstetrics, Gynecology, and Reproductive Sciences, San Francisco School of Medicine, UCSF Helen Diller Family Comprehensive Cancer Center, University of California, 1600 Divisadero Street, Box 1702, San Francisco, CA 94143, USA; 2 Division of Gynecologic Oncology, Department of Obstetrics and Gynecology, Chao Family Comprehensive Cancer Center, University of California, Irvine – Medical Center, 101 The City Drive, Orange, CA 92868, USA; 3 Department of Radiation Oncology, Stanford Cancer Center, Stanford University School of Medicine, 875 Blake Wilbur Drive, Stanford, CA 94305, USA

**Keywords:** endometrial stromal sarcoma, prognostic factors, survival

## Abstract

To determine independent prognostic factors for the survival of patients with endometrial stromal sarcoma (ESS), data were abstracted from the Surveillance, Epidemiology, and End Results (SEER) database of the National Cancer Institute from 1988 to 2003. Kaplan–Meier and Cox proportional hazards models were used for analyses. Of 831 women diagnosed with ESS, the median age was 52 years (range: 17–96 years). In total, 59.9% had stage I, 5.1% stage II, 14.9% stage III, and 20.1% had stage IV disease. Overall, 13.0, 36.1, and 34.7% presented with grades 1, 2, and 3, respectively. Patients with stage I–II *vs* III–IV disease had 5 years DSS of 89.3% *vs* 50.3% (*P*<0.001) and those with grades 1, 2, and 3 cancers had survivals of 91.4, 95.4, and 42.1% (*P*<0.001). In multivariate analysis, older patients, black race, advanced stage, higher grade, lack of primary surgery, and nodal metastasis were independent prognostic factors for poorer survival. In younger women (<50 years) with stage I–II disease, ovarian-sparing procedures did not adversely impact survival (91.9 *vs* 96.2%; *P*=0.1). Age, race, primary surgery, stage, and grade are important prognostic factors for ESS. Excellent survival in patients with grade 1 and 2 disease of all stages supports the concept that these tumors are significantly different from grade 3 tumors. Ovarian-sparing surgeries may be considered in younger patients with early-stage disease.

Endometrial stromal sarcomas (ESSs) are rare tumors of the uterus accounting for 0.2–1% of all uterine malignancies and approximately 6–20% of all uterine sarcomas (Koss *et al*, 1965; Harlow *et al*, 1986; Echt *et al*, 1990; Larson *et al*, 1990). Given the rarity of ESS, there are limited reports in the literature that have evaluated the prognostic factors of patients with this tumor. On account of significant differences in the clinical behaviour of ESS patients, Norris and Taylor first described the prognostic significance of histologic grade in ESS, and their findings have been subsequently confirmed by others (Norris and Taylor, 1966; Pautier *et al*, 2000; Leath *et al*, 2007). Furthermore, as high-grade ESS lack specific differentiation, recent modifications to pathologic classifications of uterine sarcoma have been recommended to rename high-grade ESS as undifferentiated endometrial sarcoma (Jaffe *et al*, 2001; Moinfar *et al*, 2007).

Optimal therapy of ESS is not well established. The standard surgical procedure has included a total abdominal hysterectomy, bilateral salpingo-oophorectomy, and radical cytoreductive surgery of extra-uterine disease (Gadducci *et al*, 1996). Moreover, despite traditional recommendations to include bilateral salpingo-oophorectomy in the primary surgical management of ESS, some investigators have advocated preserving ovarian function, particularly in younger women (Gadducci *et al*, 1996; Chu *et al*, 2003; Li *et al*, 2005; Amant *et al*, 2007). The role of pelvic and para-aortic lymphadenectomy, and adjuvant therapy with radiation therapy, chemotherapy, or hormonal treatment remains controversial. Most of the earlier studies have been limited by a small number of patients from academic institutions with associated biases (Echt *et al*, 1990; Haberal *et al*, 2003; Denschlag *et al*, 2007; Leath *et al*, 2007). In addition, there are no large studies that have investigated the potential risk of ovarian preservation in young patients with early-stage disease.

In this large population-based study of 831 patients, we examined the demographic and clinico-pathologic prognostic factors associated with disease-specific survival (DSS) in ESS. In addition, the role of lymphadenectomy, adjuvant radiation therapy, and oophorectomy was analyzed.

## Materials and methods

Demographic, clinico-pathologic variables, treatment data, and survival information of women diagnosed with ESS were extracted from the Surveillance, Epidemiology, and End Results (SEER) database of the United States National Cancer Institute for the 15-year period from 1 January 1988 to 31 December 2003. Data are reported from population-based registries that represent approximately 26% of the U.S. population including the states of Utah, Hawaii, Iowa, New Mexico, Connecticut, Alaska Native, and the metropolitan regions of Detroit, San Francisco-Oakland, Seattle-Puget Sound, Atlanta, San Jose-Monterey, Los Angeles, and rural Georgia. The histology code used was 8930 for ESS. Although the database coded grade of disease as 1, 2, 3 and 4, we elected to combine grades 3 and 4 as grade 3 disease given the similarity of the two groups.

Kaplan–Meier models were used to estimate 5-year DSS. Variables examined included age, race, stage, grade, primary surgery, and presence of nodal metastasis, oophorectomy, and adjuvant radiation treatment. All factors that were significant in univariate analyses were tested in the multivariable model. The Cox proportional hazards model was used to identify independent predictors of survival. All *P*-values reported are two-tailed, and a *P*-value of less than 0.05 was considered to be statistically significant.

## Results

Of 831 women with ESS, the median age at diagnosis was 52 years (range: 17–96 years). Demographic characteristics of these patients are presented in Table 1. The majority (59.9%) had stage I disease, 5.1% stage II, 14.9% stage III, and 20.1% had stage IV disease. Grades 1, 2, and 3 comprised 13.0, 36.1, and 34.7% of cases, respectively.

Analysis of surgical treatment found that 775 (93.3%) women underwent a hysterectomy; 282 (33.9%) also had a lymphadenectomy, and 28 (9.9%) women were found to have nodal metastases. Moreover, the rate of lymphadenectomy in those women 50 years and younger *vs* older than 50 years was 32.2 *vs* 35.9%, respectively. Oophorectomy was performed in 483 (58.1%) cases. In addition, 24.7% of patients underwent adjuvant radiotherapy. Table 1 provides further details of treatment characteristics. Of patients with grades 1, 2, and 3 disease, 33 (11.7%), 79 (28.0%), and 127 (45.0%) underwent lymph node dissections, of which 2 (6.1%), 7 (8.9%), and 16 (12.6%) had nodal metastases. Lastly, approximately 6.7% of the patients did not undergo primary surgery. Of these 56 patients, the median age was 68.8 years of age compared with only 51 years in those who underwent surgery. In addition, the proportion of advanced stage disease was higher in those without surgery, 82.1 *vs* 31.6%.

**Figure 1:**
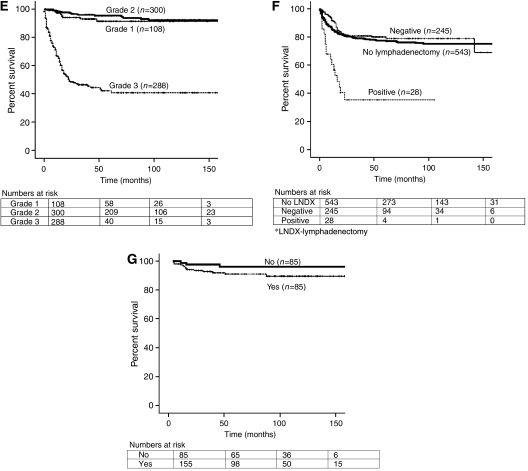
Kaplan–Meier disease-specific survival based on (**A**) age (*P*<0.001), (**B**) race (*P*=0.001), (**C**) surgery (*P*<0.001), (**D**) stage (*P*<0.001), (**E**) grade (*P*<0.001), (**F**) nodal metastasis (*P*<0.001), and (**G**) oophorectomy in younger women (<50 years) with stage I–II disease (*P*=0.1).

The 5-year DSS for the entire cohort was 76.2%. The 5-year DSS of younger patients (<52 years) was significantly higher compared with older women (85.9 *vs* 64.7%, *P*<0.001) (Table 2). Blacks had a worse DSS compared with other races (62.5 *vs* 78.1%; *P*=0.001). Those who underwent primary hysterectomy had a significant benefit over those who did not (78.5 *vs* 42.4%; *P*<0.001). Moreover, the 5-year DSS of those with stage I–II disease was 89 *vs* 50.3% in those with stages III–IV disease (*P*<0.001). Those with grades 1, 2, and 3 disease had 5-year DSSs of 91.4, 95.4, and 42.1%, respectively (*P*<0.001) (Figure 1).

In the overall study group, there was no difference in 5-year DSS in those who underwent oophorectomy *vs* ovarian-sparing procedures (*P*=0.06) (Table 2). Similarly, in the subset of younger women (<50 years) with stage I–II disease, those who underwent ovarian-sparing procedures had a similar survival compared with those who underwent oophorectomies (96.2 *vs* 91.1%; *P*=0.1). However, the exact number of patients who may have undergone adnexal surgery before their cancer diagnosis is not reported. Furthermore, lymphadenectomy and adjuvant treatment with radiation therapy had no demonstrable impact on overall survival.

Factors found to be significant in univariate analysis were then analyzed using a Cox proportional hazards model to determine independent predictors of DSS. On multivariate analysis, older age at diagnosis, black race, no surgery, advanced stage, and higher grade of disease were all independent predictors for poorer survival (Tables 3 and 4).

## Discussion

As endometrial stromal sarcomas are rare uterine malignancies, there are limited reports on their clinical behaviour and optimal treatment strategies. Most of these studies involve case reports, and retrospective case series by academic institutions with a small number of patients accrued over long time periods. In addition, others series have included additional histological subtypes of uterine sarcomas. To our knowledge, this analysis is one of the largest studies, including over 800 patients evaluated for prognostic factors important in ESS patients.

Earlier studies on the importance of age in ESS have been inconclusive. Denschlag *et al* (2007) reported on 28 patients with ESS, and found overall better survival was significantly associated with younger patient age. Similarly, Kokawa *et al* (2006) showed that younger age was an independent predictor of improved survival in multivariate analysis of 15 cases of ESS. In contrast, Nordal *et al* (1996) studied 48 patients with ESS and found that age was not significantly correlated to survival. In this study of over 800 patients, patients ⩽52 years had more than a 20% higher 5-year DSS compared with older patients. Moreover, age, as a continuous variable remained a significant prognostic factor in multivariate analysis.

In this analysis, our data also showed that blacks had a worse DSS compared with all other racial groups after adjusting for other prognostic factors. Racial and ethnic differences in treatment and survival have been previously reported for several gynecologic malignancies, including ovary and uterine cancers (Wingo *et al*, 1996; Madison *et al*, 1998; Maxwell *et al*, 2006; Morris *et al*, 2006; Chan *et al*, 2008). In uterine sarcomas, Brooks *et al* (2004) found a survival difference among racial groups, but this was no longer present after adjusting for treatment differences. In this analysis, we demonstrated that blacks with ESS have a poorer survival after adjusting for surgery and adjuvant radiotherapy. Further studies are warranted to identify the underlying cause for racial differences in survival that cannot simply be explained by treatment disparities.

Similar to other reports, our analysis found that stage and grade were important predictors of overall improved survival (Echt *et al*, 1990; Kokawa *et al*, 2006; Denschlag *et al*, 2007). In our analysis, stage I–II patients had a 5-year DSS of 89.3% compared with only 50.3% in stage III–IV disease. Grade has also long been recognized as an important factor in ESS (Norris and Taylor, 1966; Pautier *et al*, 2000; Haberal *et al*, 2003; Leath *et al*, 2007). In over 400 patients with grades 1 and 2 tumors in our study, the 5-year DSS was more than 90% compared with only 42% in those with grade 3 tumors. These findings clearly support the hypothesis that high-grade ESSs have a distinct biologic behaviour. This has also been demonstrated by others (Gadducci *et al*, 1996; Leath *et al*, 2007). In fact, there are some investigators who have recommended a change in the classification of high-grade ESS to undifferentiated endometrial sarcoma, as these tumors have distinct pathologic and clinical properties compared with low-grade ESS. The 2003 World Health Organization Classification of tumors employs this new nomenclature, as does the current pathology literature, where the term of ESS is used to describe low-grade stromal sarcomas only (Jaffe *et al*, 2001; Moinfar *et al*, 2007).

Although standard therapy for ESS has included hysterectomy and bilateral salpingo-oophorectomy, the additional extent of surgery, especially the role of pelvic and para-aortic lymphadenectomy, remains controversial. In our study, the survival of those who underwent a lymph node dissection was not significantly different compared with those without lymph node dissection (*P*=0.351). However, it was interesting to note that nearly 10% of those who underwent lymph node dissection were found to have nodal metastases. Thus, it is important to perform lymphadenectomy in ESS patients for both prognostic and treatment purposes to direct adjuvant therapy. In addition, patients with positive nodal metastasis at the time of lymphadenectomy had significantly poorer survival (35.3%) compared with those that had negative nodes (80.1%). Other reports have also suggested that the rates of lymph node involvement in ESS may be higher than expected (Goff *et al*, 1993; Reich *et al*, 2005; Riopel *et al*, 2005). Therefore, lymph node dissection clearly provides prognostic information and treatment guidance; however, the potential therapeutic value of lymph node dissection remains to be determined.

Similar to endometrioid uterine cancer, the standard recommendations have included bilateral salpingo-oophorectomy in the primary surgical management of ESS. This study showed that ovarian-sparing surgeries in the subset of younger women (<50 years) with otherwise early-stage disease did not adversely impact survival. Similarly, Li *et al* (2005) found that ovarian preservation did not affect recurrence or survival in women with stage I low-grade ESS. Similar findings were described by Amant *et al* (2007) in a multicenter analysis of 34 women with ESS. In stage I–II premenopausal women who underwent hysterectomy with or without bilateral saplingo oophorectomy, 3 of 12 (25%) and 1 of 6 (17%) recurred, respectively. Although the sample size was too small to draw conclusions, ovarian preservation did not seem to compromise outcomes. These results were also confirmed by others (Gadducci *et al*, 1996; Chu *et al*, 2003). In contrast, in a recent analysis of 53 patients, investigators found that ovarian preservation resulted in higher recurrence rates of 9 of 9 patients *vs* 10 of 44 (*P*<0.001) in those who had bilateral salpingo-oophorectomy (Li *et al*, 2008). Although our data suggest that ovarian-sparing surgeries may be considered in younger patients with early-stage disease, it is important to note that the SEER data do not include data on adnexal surgeries before cancer diagnosis. Thus, these limitations may have influenced our results.

The strength of our study is the large number of patients, which permitted subset analyses investigating the role of lymphadenectomy and oophorectomy, as well as other prognostic factors, such as age, race, and grade of disease. Moreover, the SEER database is representative of the general patient population without associated biases of case reports and studies from single academic institutions that span over many years. Several studies have demonstrated the accuracy of pathology from the SEER database (Glaser *et al*, 2001; Field *et al*, 2004). There are, however, several limitations of this study, including a lack of information regarding surgeon specialty, residual disease or margin status after primary surgery, hormone receptor status, sites of recurrence, earlier oophorectomy, chemotherapy, hormonal treatment or combined treatments.

In summary, the results of this study of 831 women with ESSs showed that age, race, stage, and grade of disease are important independent prognostic factors for survival. The survival of more than 90% in patients with grades 1 and 2 disease compared with only 42% in those with grade 3 disease supports the concept that low-grade ESSs have a significantly different clinical behaviour from high-grade tumors. The therapeutic role of lymphadenectomy and adjuvant therapy remains unclear. However, our data suggest that ovarian-sparing surgeries may be considered in younger patients with early-stage disease.

## Figures and Tables

**Table 1:** Demographic and clinico-pathologic characteristics (*n*=831)

**Characteristics**	**n**	**%**
*Age at diagnosis (years)*		
Median age (range)	52 (17–96)	
⩽52 years	432	52.0
>52 years	399	48.0
		
*Year of diagnosis*		
1988–1992	150	18.0
1993–1997	284	34.2
1998–2003	397	47.8
		
*Race*		
White	553	66.5
Black	114	13.7
Hispanic	82	9.9
Asian	70	8.4
Other	12	1.4
		
*Stage*		
I	498	59.9
II	42	5.1
III	124	14.9
IV	167	20.1
		
*Grade*		
1	108	13.0
2	300	36.1
3	288	34.7
Unknown	135	16.2

**Table 2:** Treatment characteristics (*n*=831)

**Characteristics**	**n**	**%**
*Surgery*		
Yes^a^	775	93.3
No	56	6.7
		
*Lymphadenectomy*		
Yes	282	33.9
Positive	28	9.9^b^
Negative	245	86.9^b^
Unknown	9	3.2^b^
No	543	65.3
Unknown	6	0.7
		
*Oophorectomy*		
Yes	483	58.1
No	290	34.9
Unknown	58	7.0
*Age <50 years, stage I–II*		
Yes	155	64.6^c^
No	85	35.4^c^
		
*Radiation*		
Yes	205	24.7
No	611	73.5
Unknown	15	1.8

a Primary hysterectomy.

b Percent of those undergoing lymphadenectomy.

c Percent of those <50 years, stage I–II.

**Table 3:** Five-year disease-specific survival based on demographic and clinico-pathologic prognostic factors (*n*=831)

**Characteristic**	**%**	***P-*value**
*Overall*	76.2 (±1.6)	
*Age at diagnosis (years)*		*P*<0.001
⩽52	85.9 (±1.8)	
>52	64.7 (±2.6)	
		
*Year of diagnosis*		*P*=0.008
1988–1992	72.2 (±3.7)^a^	
1993–1997	83.9 (±2.2)^a^	
1998–2003	74.4 (±2.4)^a^	
		
*Race*		*P*=0.005
White	77.1 (±1.9)	
Black	62.5 (±5.0)	
Hispanic	79.6 (±4.7)	
Asian	83.8 (±5.1)	
		
*Surgery*		*P*<0.001
Yes^b^	78.5 (±1.6)	
No	42.4 (±7.4)	
		
*Oophorectomy*		*P*=0.06
Yes	78.1 (±2.0)	
No	72.9 (±2.7)	
*Age <50 years, stage I–II*		*P*=0.1
Yes	91.1 (±2.4)	
No	96.2 (±2.1)	
		
*Stage*		*P*<0.001
I	91.7 (±1.3)	
II	52.8 (±9.9)	
III	61.5 (±4.8)	
IV	41.0 (±4.4)	
I–II	89.3 (±1.4)	*P*<0.001
III–IV	50.3 (±3.3)	
		
*Grade*		*P*<0.001
1	91.4 (±3.0)	
2	95.4 (±1.3)	
3	42.1 (±3.8)	
		
*Lymphadenectomy*		*P*=0.351
Yes	73.8 (±2.9)	
No	77.6 (±1.9)	
		
*Nodal metastasis*		*P*<0.001
Positive	35.3 (±9.6)	
Negative	80.1 (±2.8)	

a 3-year disease-specific survival.

b Primary hysterectomy.

**Table 4:** Multivariate analysis

**Prognostic factor**	**Hazard ratio**	**95% Confidence interval**	***P*-value**
Age at diagnosis^a^	1.02	1.01–1.03	*P*<0.007
Race^b^	1.70	1.18–2.45	*P*=0.013
Surgery^c^	0.36	0.23–0.57	*P*<0.001
Stage^d^	1.99	1.73–2.28	*P*<0.001
Grade^e^	9.04	5.77–14.17	*P*<0.001

a Continuous.

b Non-black *vs* black.

c No primary hysterectomy *vs* primary hysterectomy.

d I *vs* II *vs* III *vs* IV.

e 1 *vs* 2 *vs* 3.
